# Evaluating and comparing the predictive ability with hypertension risk among obesity indicators: a prospective cohort integrating Mendelian randomization analysis

**DOI:** 10.3389/fnut.2025.1660842

**Published:** 2025-12-05

**Authors:** Fengling Wang, Yixiao Wang, Han Pang, Hui Zhang, Shouzheng Wei, Zhuang Zhuo, Zihan Liu, Mu Wang, Chongjian Wang

**Affiliations:** 1 Department of Preventive Medicine, School of Public Health, Gansu University of Chinese Medicine, Lanzhou, Gansu, China; 2 Department of Epidemiology and Biostatistics, College of Public Health, Zhengzhou University, Zhengzhou, Henan, China; 3 Department of Clinical Medicine, First Affiliated Hospital of Zhengzhou University, Zhengzhou, China; 4 Clinical Mass Spectrometry Laboratory, Clinical Research Institute, Affiliated Nanhua Hospital, Hengyang Medical School, University of South China, Hengyang, Hunan, China

**Keywords:** obesity-related indicator, hypertension, predictive ability, Mendelian randomization, rural population

## Abstract

**Objective:**

This study aimed to compare and evaluate the predictive ability of obesity-related indicators for new-onset hypertension, and to explore causal effects using Mendelian randomization (MR).

**Methods:**

A total of 22, 912 eligible participants were included from the Henan Rural Cohort Study. Logistic regression was used to identify key predictors of hypertension, and gradient boosting machine (GBM) models incorporating ten obesity indices were developed. Model performance was evaluated using the area under the curve (AUC), and indicator importance was assessed with SHapley Additive exPlanations (SHAP). A multi-state Markov model estimated life expectancy (LE) and health-adjusted life expectancy (HALE). Causal associations were examined through MR analysis.

**Results:**

The basic GBM model achieved an AUC of 0.835 (95% *CI*: 0.826–0.844), with body mass index (BMI) showing the highest predictive value (AUC = 0.844). SHAP analysis revealed that all obesity indicators were positively associated with new-onset hypertension but ranked below age and blood pressure level in importance. At age 18, LE was 62.72, 66.11, 68.79 years for individuals with normal weight, overweight, and obesity, respectively. The corresponding HALE was 29.45, 26.23, 22.29 years. MR analysis confirmed causal associations of obesity indicators with hypertension.

**Conclusion:**

Obesity-related indicators are significantly associated with new-onset hypertension, and they are linked to increased LE and reduced HALE. The findings provide evidence to early recognition and prevention of hypertension risk based on obesity-related indicators.

## Highlights

- This is the first study to compare the predictive ability of obesity-related indicators with hypertension.
- BMI demonstrates the strongest predictive ability with new-onset hypertension.
- Higher obesity levels are associated with increased LE and reduced HALE.
- MR analysis confirms obesity-related indicators have a positive causal link with hypertension.
- This study offers implications for early recognition of hypertension risk by obesity-related indicators.

## 1 Introduction

Hypertension, defined as systolic blood pressure (SBP) ≥ 140 mmHg and/or diastolic blood pressure (DBP) ≥ 90 mmHg without antihypertensive medication, affects approximately 274 million individuals in China, with a prevalence rate of 24.7%. Globally, hypertension contributes to 8.5 million deaths from stroke, ischemic heart disease, and other related conditions (1). However, the awareness and control rates of hypertension remain low in rural areas (2). Identifying risk factors and implementing effective prevention and intervention strategies are crucial.

Obesity is a well-established independent risk factor for hypertension (3). However, traditional body mass index (BMI) does not fully capture fat distribution or differentiate between body composition. To overcome these limitations, other obesity indicators such as waist circumference (WC), waist-to-hip ratio (WHR), waist-to-height ratio (WHtR), body fat percentage (BFP), and visceral fat index (VFI) have been proposed (4). Furthermore, emerging indicators like lipid accumulation product (LAP), body roundness index (BRI), and a body shape index (ABSI) have also been examined for their association with hypertension risk (5–7). Despite these advancements, there remains controversy regarding which obesity indicator is most strongly associated with hypertension.

Gradient boosting machine (GBM), a method in machine learning, iteratively fits new models to improve accuracy in estimating response variables (8). However, research applying machine learning to construct models and assess the effectiveness of various obesity indicators in predicting hypertension is limited. SHapley Additive exPlanation (SHAP) is a technique that assigns specific importance values to each variable, helping to elucidate their roles in predictive models (9–11). In this study, we aimed to establish predictive models using GBM and SHAP to assess and compare the strength of associations between ten different obesity indicators and the incidence of hypertension.

Although it is widely believed that being overweight or obese increases the risk of hypertension (12–14), many observational studies are subject to reverse causation and residual confounding. To address these limitations, we employed Mendelian randomization (MR), a method that utilizes genetic variants associated with the exposure of interest to examine the effect of changing this exposure on disease risk. In this study, we used genome-wide significant single nucleotide polymorphisms (SNPs) retrieved from large-scale genome-wide association studies (GWAS) as instruments for two-sample MR to assess the causal relationship between hypertension and four anthropometric measures, including BMI, WC, WHR, and BFP.

In this study, we utilized data from the Henan Rural Cohort Study to compare the predictive strength of ten obesity indicators for the risk of hypertension using a machine learning approach. In addition, life expectancy (LE) and health-adjusted life expectancy (HALE) were estimated based on obesity-related indicators and new-onset hypertension in order to provide potential evidence for the ‘obesity paradox’. We also explored potential causal relationships through MR analysis. This research aimed to provide new insights into primary prevention strategies and to contribute to the alleviation of the hypertension burden, particularly in rural populations.

## 2 Methods

### 2.1 Study population

Participants in the study were drawn from the Henan Rural Cohort Study, conducted in Suiping, Tongxu, Xinxiang, Yima, and Yuzhou, five rural counties within Henan Province. Detailed information has been previously reported (15, 16). A total of 39, 259 participants aged 18 years and older were included in this study. Participants were excluded from the analysis if they met any of the following criteria: ① were dead or lost to follow-up (*n* = 4, 270); ② were diagnosed with hypertension at baseline (*n* = 11, 201); ③ had missing information on hypertension at follow-up (*n* = 692); or ④ were diagnosed with cancer or kidney failure at baseline (*n* = 184). After excluding, a total of 22, 912 participants remained in the study cohort. Given the low proportion of missing data, subjects with missing covariates or the specific obesity indicator were directly excluded from each respective model. The research has been approved by the Zhengzhou University Life Science Ethics Committee and conducted in accordance with the principles of the Declaration of Helsinki. All participants provided written informed consent before the study commenced. To ensure adequate statistical power, we not only estimated the sample size based on the different obesity indicators, but also assessed the sample size and power analysis in AUC-based model validation using the Hanley-McNeil method. The current study ultimately included 22,801 participants, substantially exceeding the sample size estimates and ensuring sufficient statistical power. The detailed process and approaches were presented in Text S1.

### 2.2 Measurements

Anthropometric measurements including weight, height, WC, hip circumference, BFP and VFI were carefully measured using standardized protocols (17). Body weight was measured using a weight-measuring device (accurate to 0.1 kg). BFP and VFI were measured with subjects in light clothing and without shoes, using the OMRON V. BODY HBF-371 device, following the manufacturer’s instructions. Height was measured without shoes (accurate to 0.1 cm). For WC measurement, participants wore lightweight clothing, and the measurement was taken to an accuracy of 0.1 cm. BMI < 24.0 kg/m^2^ was classified as normal, 24.0–27.9 kg/m^2^ as overweight, and ≥28.0 kg/m^2^ as obese. The WC cutoff was 85 cm for men and 80 cm for women (18). WHR cutoffs were 0.9 for men and 0.8 for women, and WHtR cutoff was 0.5 (19). LAP, BRI, ABSI and BAI were calculated as follows: LAP: [WC (cm) − 65] × TG (mmol/L) for men and [WC (cm) − 58] × TG (mmol/L) for women (20). BRI: BRI = 364.2–365.5 × {1 − [WC (m)/2π/0.5 × height (m)]^2^}^1/2^ (21). ABSI: ABSI = WC (m)/ (BMI^2^/3 × height (m)^1/2^) (22). BAI: BAI = [HC (cm)/height (m)^1.5^] − 18 (23). BFP, VFI, LAP, BRI, ABSI and BAI were grouped into tertiles.

The resting blood pressure was measured three times using an electronic sphygmomanometer (Omron HEM-7071A, Japan) on the right arm supported at a heart-level while seated. The average of these three readings was used for analysis. Baseline hypertension was defined based on Chinese guidelines as systolic blood pressure ≥140 mmHg and/or diastolic blood pressure ≥90 mmHg at baseline measurement. Participants who had received a hypertension diagnosis from a physician and were taking antihypertensive medication within the past 2 weeks were also classified as hypertensive. Incident hypertension was defined as participants who were free of hypertension at baseline but were identified as hypertensive during follow-up according to the same criteria described above.

A structured questionnaire was used to collect sociodemographic information including age, sex, marital status, education level, monthly income. Lifestyle factors included smoking status, drinking status, physical activity, high-fat diet (≥ 75 g/day), and vegetable and fruit intake (≥ 500 g/day) (24). Current smoking was defined as consuming at least one cigarette per day over the past 6 months, while current alcohol consumption was defined as having consumed at least 12 drinks in the past year. Physical activity levels were classified according to the International Physical Activity Questionnaire (IPAQ) (25). All information in the questionnaire was self-reported and collected by trained investigators.

### 2.3 Mendelian randomization analysis

Four anthropometric variables, including BMI, WC, WHR, and BFP, were categorized as exposures, while hypertension, systolic blood pressure (SBP), and diastolic blood pressure (DBP) were considered outcomes. The MR method requires satisfying three fundamental instrumental variables (IVs) assumptions (Supplementary Figure S1) (26). To meet the first assumption, we selected genome-wide significant (*p* ≤ 5 × 10^−8^) SNPs as IVs; however, directly testing the other assumptions is challenging, thus we conducted a series of sensitivity analyses to detect pleiotropy among IVs and validate our findings (Supplementary Figure S2). The data utilized in our study are publicly available, with participants from GWAS studies primarily of European ancestry. The hypertension GWAS comprises 54,358 positive cases and 408, 652 controls of European ancestry. Data on WC, WHR, DBP, and SBP were extracted from the UK Biobank, a prospective cohort study involving over half a million individuals aged 40–69 years in the UK (27) (Supplementary Table S1).

To select eligible IVs from summary-level data, we conducted the following five-step quality control process: ① Genome-wide significant SNPs (*p* ≤ 5 × 10^−8^) associated with four anthropometric variables were chosen as candidate IVs; ② SNPs with minor allele frequencies less than 0.01 were excluded; ③ SNPs with alleles incompatible with the outcome were excluded; ④ Palindromic variants with ambiguous strand were excluded; ⑤ Lead SNPs were collected from the selected genetic variants to ensure independence of IVs, using parameters (window size = 10, 000 kb, *r^2^* < 0.001) (28). To address the potential issue of population mismatch (the original GWAS data were primarily from European populations, whereas our study cohort is Chinese), we conducted an additional sensitivity MR analysis using GWAS data from East Asian populations. The exposure variable was BMI, and the outcome variable was hypertension. We selected SNPs significantly associated with BMI in the East Asian GWAS at a threshold of *p* ≤ 1 × 10^−5^, and performed clumping (*r^2^* < 0.001, window size 10,000 kb) to obtain independent instrumental variables.

### 2.4 Statistical analysis

Binary logistic regression was used to analyze the relationship between various obesity indicators and the risk of hypertension. Additionally, a restricted cubic spline (RCS) model was employed to explore the dose–response relationship between obesity indicators and blood pressure. Multivariable logistic regression was used to select variables for building the prediction model. Age, gender, vegetables and fruit intake, salt-intake, physical activity, SBP, DBP, income and family history of hypertension were ultimately included as covariates in the models. GBM was employed to develop models for the incidence risk of hypertension. The model without any obesity indicators included was used as the basic model, and different obesity indicators were incorporated into this basic model sequentially. The AUC of the prediction models was calculated to compare their predictive performance, and SHAP was utilized to explain their importance as predictor variables. Reclassification capability was quantified through the net reclassification improvement (NRI) and integrated discrimination improvement (IDI), and calibration was evaluated using the Brier score to measure the agreement between predicted probabilities and actual outcomes. Subgroup analyses were performed by age (<60 vs. ≥60 years), gender (male vs. female), salt-intake (high-salt diet vs. non-high-salt diet), and physical activity (low vs. moderate vs. high). Additionally, we implemented the multistate life table method to calculate the LE and HALE. This method assumes that different health states are non-transmutable and constructs a matrix based on transition probabilities between different states derived from dynamic follow-up data. The Markov multistate transition model, which includes three states (non-hypertension, hypertension, and death), was fitted using the “msm” package. Three types of transitions were considered: ① from non-hypertension to hypertension; ② from hypertension to death; ③ from non-hypertension to death. Age was included as a time-dependent covariate, and the model was additionally adjusted for gender, income, vegetable and fruit intake, physical activity, and different obesity indicators.

We employed several MR methods to investigate potential causal relationships between anthropometric measures and hypertension, SBP, or DBP. Initial analysis utilized the inverse variance weighted (IVW) method to ensure precise estimation of summary-level data (29). To mitigate the impact of pleiotropy, three MR methods with different assumptions were used as sensitivity analyses. MR-PRESSO was used to identify and correct for horizontal pleiotropy, removing outliers to eliminate the influence of pleiotropy (30). Sensitivity analyses included the weighted median, MR-Egger, and MR-PRESSO methods. Statistical analyses were performed using SPSS 21.0 and R software (version 4.3.2).

## 3 Results

### 3.1 Characteristics of the population

During a median follow-up period of 4.0 years, 1, 655 (7.22%) participants developed hypertension, and general characteristics of the participants were presented in Table 1. The comparison results between two groups showed that ten obesity indicators were significantly higher in the hypertensive group compared to the non-hypertensive group (*p <* 0.001; details provided in Supplementary Table S2). In addition, several obesity indicators were categorized into tertiles, with the corresponding cutoff values reported in Supplementary Table S3.

**Table 1:** Basic characteristics of participants according to hypertension.

Variables	Total (*n* = 22, 912)	Hypertension (*n* = 1, 655)	Non-hypertension (*n* = 21, 257)	*p* value
Age (years)	52.86 ± 12.26	57.85 ± 10.74	52.47 ± 12.28	<0.001
Women	14,030 (61.23)	1,019 (61.57)	13,011 (61.21)	0.793
Married/cohabitating	21,010 (91.70)	1,479 (89.37)	19,531 (91.88)	<0.001
Education level				
Elementary school or below	9,115 (39.78)	810 (48.94)	8,305 (39.07)	<0.001
Junior high school	9,837 (42.94)	614 (37.10)	9,223 (43.39)	
High school or above	3,960 (17.28)	231 (13.96)	3,729 (17.54)	
Average monthly income				<0.001
<500 CNY	7,631 (33.31)	639 (38.61)	6,992 (32.90)	
500 ~ CNY	7,554 (32.97)	534 (32.27)	7,020 (33.02)	
≥1,000 CNY	7,727 (33.72)	482 (29.12)	7,245 (34.08)	
Physical activity				0.043
Low	6,667 (29.10)	505 (30.51)	6,162 (28.98)	
Moderate	9,155 (39.96)	683 (41.27)	8,472 (39.86)	
High	7,090 (30.94)	467 (28.22)	6,623 (31.16)	
Current smokers	4,691 (20.47)	326 (19.70)	4,365 (20.53)	0.429
Current drinkers	4,207 (18.36)	305 (18.43)	3,902 (18.36)	0.950
High-fat diet	4,824 (21.05)	310 (18.73)	4,514 (21.24)	0.017
More vegetables and fruits intake	10,373 (42.28)	772 (46.65)	9,601 (45.17)	0.249
High-salt diet	4,038 (17.64)	315 (19.03)	3,723 (17.54)	0.123
Baseline SBP (mmHg)	115.48 ± 11.80	125.90 ± 8.74	114.67 ± 11.62	<0.001
Baseline DBP (mmHg)	72.46 ± 8.10	77.74 ± 7.23	72.05 ± 8.02	<0.001
Family history of hypertension	3,540 (15.45)	257 (15.53)	3,283 (15.44)	0.918

Data were presented as n (%) or mean **±** standard deviation (SD), as appropriate.

CNY, Chinese Yuan; SBP, systolic blood pressure; DBP, diastolic blood pressure.

### 3.2 Obesity indicators and new-onset hypertension

**Figure 1:**
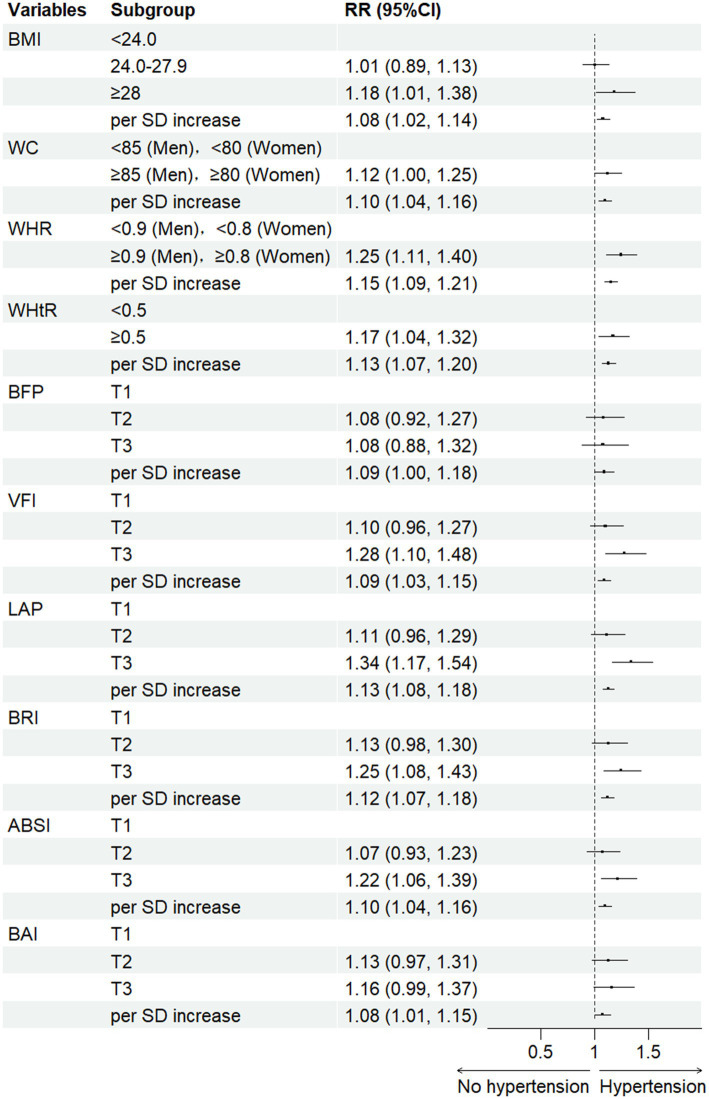
*RRs* of hypertension for different obesity indicators using logistic regression. Model adjusted for age, gender, smoking status, more vegetable and fruit intake, family history of hypertension, SBP and DBP at baseline.

Figure 1 presents the relationship between obesity indicators and hypertension risk. In the adjusted models, most obesity indicators exhibited a statistically significant association with hypertension risk when comparing the third and first quartiles (*p <* 0.05). Among these indicators, LAP demonstrated the strongest association with hypertension, with the highest relative risk (*RR* = 1.34, 95% *CI*: 1.17–1.54) observed in the third quartile. Furthermore, individuals in the highest category of obesity indicators had a significantly higher risk of hypertension compared to the reference group. Additionally, we used RCS curves to assess the dose–response relationship between standardized obesity indicators and blood pressure was further evaluated (Supplementary Figure S3). The curves showed that higher obesity indicators are correspond to elevated blood pressure levels.

Table 2 presents the AUC values for the basic model and models incorporating different obesity indicators. The AUC of the basic model was 0.835 (95% *CI*: 0.826–0.844). Incorporation of various obesity indicators increased the AUC, ranging from 0.835 to 0.844. Among these models, the BMI model exhibited the highest predictive ability. Supplementary Table S4 presents the discrimination and calibration capability evaluation of predictive models developed using GBM, including NRI, IDI, and Brier Score, and the results showed that BMI and BFP demonstrated excellent discrimination and calibration performance. Supplementary Figure S4 presents the receiver operating characteristic (ROC) curves for the basic model and models incorporating different obesity indicators. The results of subgroup analyses were consistent with those of the main analysis, with the model incorporating BMI yielding relatively higher AUC values (Supplementary Tables S5–S13).

**Table 2:** AUC of predictive models established by GBM.

Model	AUC	95% *CI*
Basic model	0.835	0.826–0.844
Basic model + BMI	0.844	0.835–0.853
Basic model + WC	0.841	0.832–0.850
Basic model + WHR	0.840	0.831–0.849
Basic model + WHtR	0.841	0.832–0.850
Basic model + BFP	0.842	0.833–0.850
Basic model + VFI	0.839	0.830–0.848
Basic model + LAP	0.841	0.832–0.850
Basic model + BRI	0.842	0.833–0.851
Basic model + ABSI	0.835	0.826–0.844
Basic model + BAI	0.839	0.831–0.849

AUC, area under the curve; GBM, gradient boosting machine.

**Figure 2:**
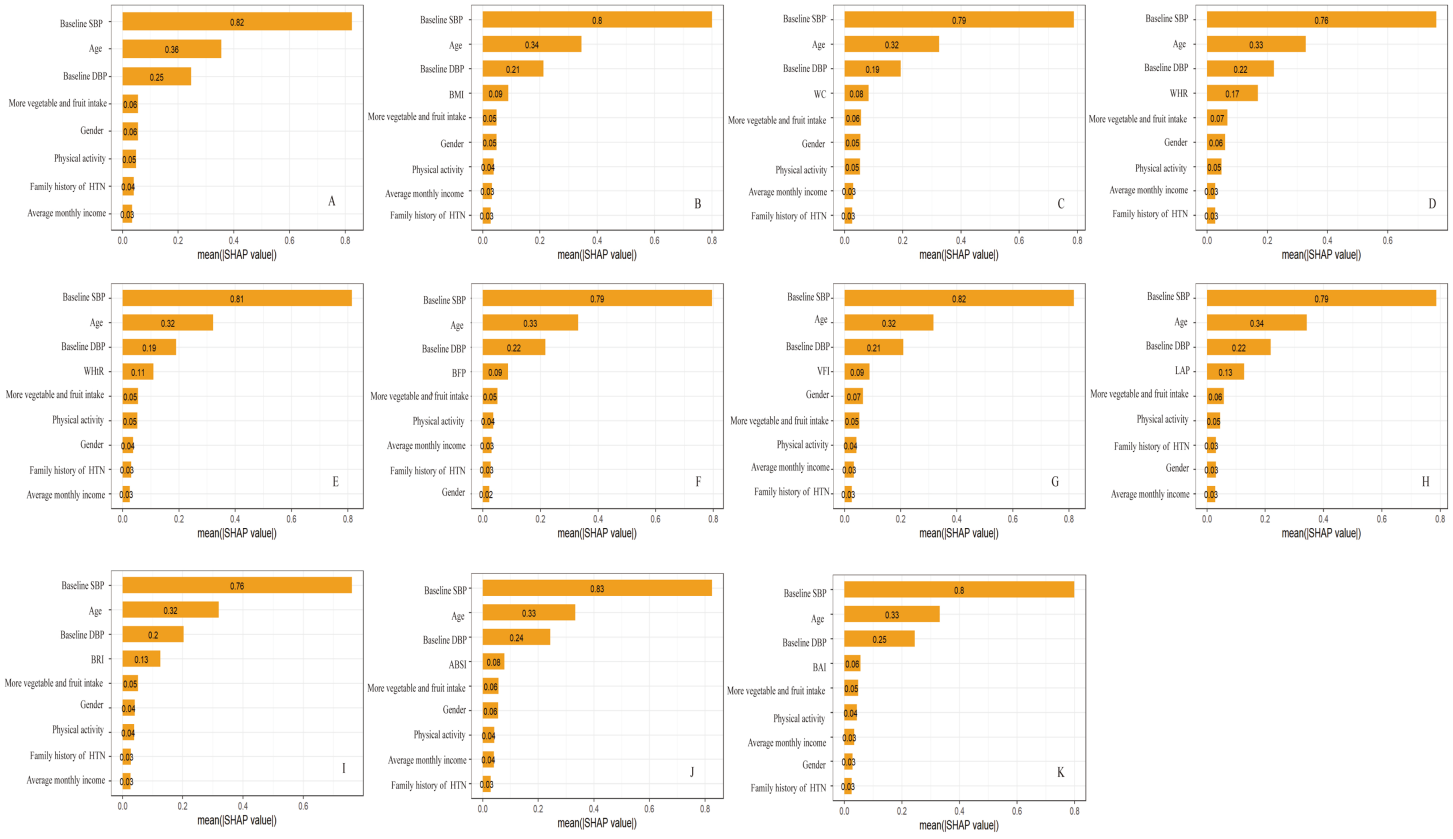
The rankings of SHAP values before and after the inclusion of obesity indicators. **(A)** basic model; **(B)** basic model + BMI; **(C)** basic model + WC; **(D)** basic model + WHR; **(E)** basic model + WHtR; **(F)** basic model + BFP; **(G)** basic model + VFI; **(H)** basic model + LAP; **(I)** basic model + BRI; **(J)** basic model + ABSI; **(K)** basic model + BAI. SHAP, SHapley Additive exPlanation.

The SHAP method was used to assess the relative importance of the variables included in the GBM model. In the basic model, the top five variables were baseline SBP, age, DBP, more vegetable and fruit intake, and gender. When obesity indicators were added, each of them was found to occupy the fourth position (Figure 2). The median SHAP values and interquartile (IQR) ranges for all obesity indicators are reported in Supplementary Table S14.

We further compared the BMI distributions of participants classified by the GBM model as low-risk versus high-risk for hypertension. The mean BMI was 23.9 kg/m^2^ in the low-risk group and 25.3 kg/m^2^ in the high-risk group; detailed values for the other obesity indicators are provided in Supplementary Table S15.

### 3.3 Estimated life expectancy

The association between obesity indicators and estimated life expectancy at 18 years old using the multi-state Markov model was examined. As obesity indicators increase, participants exhibited longer total LE, shorter HALE, and a lower ratio of HALE/LE. In the BMI-classified groups of normal weight, overweight, and obese, LE with hypertension increased, with values of 33.27, 39.88, and 46.50. Conversely, HALE at 18 years of age showed a decreasing trend across the groups, with values of 29.45, 26.23, and 22.29, respectively (Table 3). Apart from ABSI, other obesity indicators exhibited trends similar to BMI (Supplementary Figure S5).

**Table 3:** HALE, LE and HALE/LE at 18-year-old.

Indicators	HALE	LE	HALE/LE
BMI			
< 24.0	29.45	62.72	0.47
24.0–27.9	26.23	66.11	0.40
≥ 28.0	22.29	68.79	0.32
WC			
< 85 (Men), < 80 (Women)	30.63	63.56	0.48
≥ 85 (Men), ≥ 80 (Women)	24.56	66.27	0.37
WHR			
< 0.9 (Men), < 0.8 (Women)	32.29	64.11	0.50
≥ 0.9 (Men), ≥ 0.8 (Women)	24.00	65.86	0.36
WHtR			
< 0.5	32.40	62.58	0.52
≥ 0.5	24.68	66.16	0.37
BFP			
T1	37.93	65.81	0.58
T2	31.13	65.38	0.48
T3	23.47	65.10	0.36
VFI			
T1	31.93	63.09	0.51
T2	26.05	65.33	0.40
T3	19.45	67.32	0.29
LAP			
T1	32.03	61.12	0.52
T2	27.86	64.57	0.43
T3	22.79	67.52	0.34
BRI			
T1	33.23	63.44	0.52
T2	28.31	64.87	0.44
T3	22.65	66.17	0.34
ABSI			
T1	32.39	67.65	0.48
T2	27.41	65.79	0.42
T3	22.35	63.78	0.35
BAI			
T1	33.46	64.09	0.52
T2	29.30	65.02	0.45
T3	24.47	65.77	0.37

HALE, hypertension-free life expectancy; LE, life expectancy; HALE/LE, proportion of HALE to LE.

### 3.4 Causal relationships of obesity indicators and hypertension

**Figure 3:**
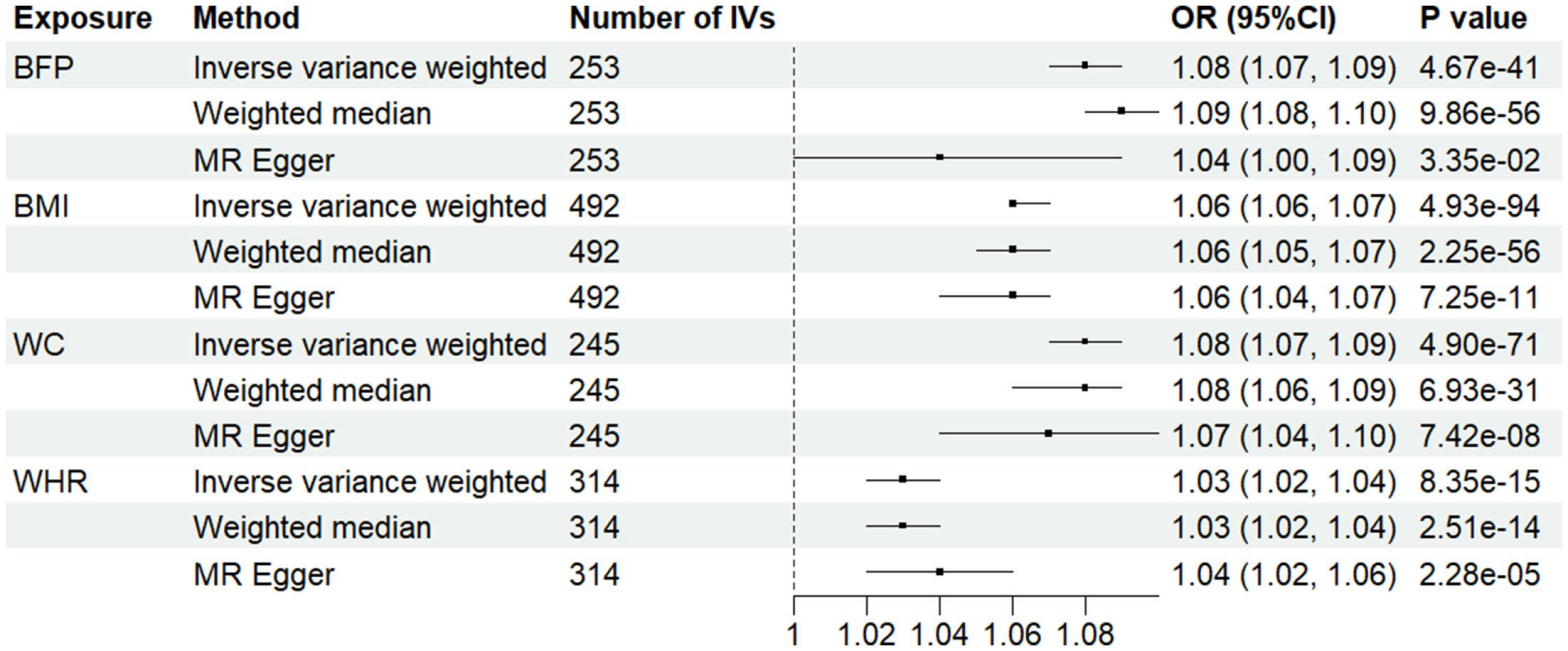
Associations between anthropometric indicator and hypertension in Mendelian randomization analyses. MR-Egger, Mendelian randomization-egger; *CI*, confidence interval; OR, odds ratio.

Figure 3 depicts the MR results for four obesity indicators in relation to hypertension. We identified causal effects of BFP, BMI, WC, and WHR on hypertension risk using the IVW method: the odds ratios (*OR*) and 95% *CI* were 1.08 (1.07, 1.09), 1.06 (1.06, 1.07), 1.08 (1.07, 1.09), and 1.03(1.02, 1.04), respectively. This indicates that each SD increase in BFP, BMI, WC, and WHR is associated with an 8, 6, 8, and 3% higher risk of hypertension. We identified causal effects of BFP, BMI, WC, and WHR on SBP and DBP (Supplementary Table S16).

The statistics from IVW and MR-Egger analyses indicate heterogeneity in the effects of the four obesity indicators on hypertension, SBP, and DBP, so a random effects model was used (Supplementary Table S17). MR-Egger intercept tests indicated evidence of horizontal pleiotropy for BMI in relation to DBP (Supplementary Table S18). MR-PRESSO analysis, which corrected for outliers, did not substantively alter the original findings (Supplementary Table S16).

To address potential population mismatch between the European GWAS and our Chinese cohort, we performed a sensitivity MR analysis using East Asian GWAS summary statistics for BMI. The causal estimate of BMI on hypertension was significant using the IVW method: the odds ratio (*OR*) and 95% *CI* were 1.44 (1.07, 1.93) (Supplementary Table S16). Heterogeneity tests showed no significant heterogeneity (Supplementary Table S17). Horizontal pleiotropy assessed by the MR-Egger intercept test indicated that the causal estimate was unlikely to be biased by directional pleiotropy (Supplementary Table S18). Overall, these results support the robustness of the causal association between BMI and hypertension in an East Asian population, mitigating concerns about population stratification bias.

## 4 Discussion

In this large prospective study, all ten obesity indicators were significantly associated with an increased risk of new-onset hypertension. The GBM-based model showed improved performance for hypertension risk after incorporating various obesity indicators, with BMI exhibiting the highest ROC. Additionally, individuals with higher body weight had a significantly shorter hypertension-free life expectancy than those with normal weight, a trend observed across all indicators except ABSI. MR analysis provided evidence of a positive causal relationship between WC, BMI, and WHR and hypertension, with findings supported by the absence of horizontal pleiotropy and outliers.

This study employed GBM to investigate the strength of association between different obesity indicators and hypertension risk among Chinese rural residents. The link between obesity and increased hypertension risk has been extensively studied. A cross-sectional survey conducted in Changsha found that obese individuals had a significantly higher risk of hypertension compared to those with normal body weight (OR: 2.60, 95% CI: 1.84–3.66) (31). A study on middle-aged and elderly individuals in China found that controlling BMI and WHR can effectively prevent and manage hypertension. Obesity shows evidence of adrenergic activation, which are linked to hypertension. Leptin regulates appetite and energy expenditure via central nervous system receptors. Leptin deficiency can cause hyperphagia and obesity (32).

This study found that BMI has the strongest association with incident hypertension. Recent large-scale studies have also highlighted the importance of adiposity indicators in hypertension. Evidence from South Brazil suggested that BMI had the largest areas under the ROC curve relative to hypertension (33). Comparative analyses in Chinese cohorts also reported that novel indices BRI and ABSI are associated with new-onset hypertension (34). Other studies in China found that the utility of WHtR and Tyg-WHtR are cost-effective indicators with moderate predictive value for hypertension (35). In Mauritian Indian women, BMI is a stronger predictor of hypertension cases than WHR (*p* = 0.047) (36). A nationwide cross-sectional study from Albania found that BMI outperformed WC and CI in predicting hypertension in both sexes (37). The findings from these studies are not entirely consistent, which may reflect heterogeneity across populations in different regions and ethnic groups. Our study therefore contributes a unique perspective by focusing on rural Chinese residents, a population that has been underrepresented in previous research.

BMI is a simple indicator used to assess overall body fat and is a measure of relative weight. It is not limited to fat in any specific area of the body, thus providing a more comprehensive assessment of body fat storage and associated metabolic issues, such as insulin resistance and inflammation, which are closely related to hypertension. Although the addition of BMI to the baseline model resulted in a statistically significant increase in AUC from 0.835 to 0.844, the AUCs of other obesity-related parameters were also relatively high (≥0.835), and the absolute improvement was modest. This modest gain likely reflects the fact that the baseline model already incorporated multiple strong predictors, leaving limited incremental predictive value for BMI. Consequently, while the improvement is statistically significant, its clinical impact may be limited, particularly in terms of influencing individual risk stratification or management decisions. Moreover, because multiple obesity indicators were evaluated, the risk of multiplicity arises; thus, the statistical significance of any single comparison should be interpreted cautiously. Our study also demonstrated that participants classified by the GBM model into the high-risk group for hypertension had a mean BMI of 25.0 kg/m^2^, compared with 23.7 kg/m^2^ in the low-risk group. This suggests that, within our rural Chinese cohort, the critical BMI range that distinguishes elevated from lower hypertension risk may lie around 24–25 kg/m^2^, which is notably lower than the clinical obesity threshold of 28.0 kg/m^2^ defined by current Chinese guidelines, and also lower than the WHO international standard of 30.0 kg/m^2^ for obesity. This discrepancy highlights that data-driven, population-specific thresholds may provide more sensitive risk stratification than conventional cut-offs, especially in populations with distinct body composition characteristics. Nevertheless, it must be emphasized that this cut-off is conditional and warrants validation in an independent external cohort.

In this study, we observed a paradoxical trend in which higher body weight was associated with longer LE, a phenomenon consistent with the so-called “obesity paradox” (38). This paradox refers to the counterintuitive observation that, although obesity is strongly linked to higher morbidity and mortality, obese individuals in certain patient groups show lower mortality and morbidity than those with normal weight. Our findings similarly demonstrated that while individuals with obesity had an increased incidence of hypertension, their overall LE was longer. However, analysis of HALE indicated that the obese population experienced a shorter duration of life free of hypertension, suggesting that the observed increase in LE may be largely attributable to longer survival after disease onset. Therefore, although obesity may be associated with increased longevity in this cohort, it concurrently reduces healthy life expectancy and diminishes overall quality of life. However, we note that ABSI displayed a pattern distinct from other obesity indicators in relation to LE. ABSI is based on WC, weight, and height, where high ABSI indicates that WC is higher than expected for a given height and weight and corresponds to a more central concentration of body volume. It could capture body shape and relative central adiposity rather than overall body mass. Therefore, ABSI may better reflect disproportionate central/visceral adipose tissue (VAT) relative to body size. Increased VAT is biologically active, producing pro-inflammatory adipokines and promoting insulin resistance, activation of the renin–angiotensin–aldosterone system and sympathetic nervous system, endothelial dysfunction, and accelerated vascular aging-pathways that plausibly increase mortality and reduce healthy lifespan (39, 40). However, regarding the results of life expectancy, we have not yet conducted a formal external validation. Additionally, the median follow-up time of this cohort is relatively short (approximately 4.0 years), which limits the direct observation of long-term mortality. Under such circumstances, our results are primarily intended for comparing the relative differences between different exposure groups, rather than serving as definitive conclusions on the absolute life expectancy of the general population.

In this study, we employed several advanced MR methods to investigate whether BFP, BMI, WC, and WHR are risk factors for hypertension, SBP or DBP. Results from the IVW method provided genetic evidence indicating a positive correlation and causal relationship of obesity indicators with hypertension, SBP or DBP. The consistency of estimates from different analytical approaches strongly supports obesity as a causal factor for hypertension. These findings are consistent with previous research demonstrating the causal relationship between obesity and hypertension (41–43).

This study has several strengths. Firstly, to our knowledge, it is the first to explore the predictive ability of different obesity indicators for hypertension using the GBM modeling method. Secondly, this study calculated LE without hypertension and under different obesity indicators, investigating the effects of varying body weights on both longevity and quality of life. The data utilized in this study were derived from the Rural Cohort Study in Henan Province. Additionally, the cohort had a substantial sample size, and a comprehensive array of potential confounding variables were collected, enhancing the reliability of the findings. By employing MR analysis, our study mitigated confounding factors and addressed issues of reverse causation. However, it is important to note several limitations. First, participants were recruited from five rural areas in Henan Province, which may limit the generalizability of our findings. Population-specific factors including environmental exposures, dietary patterns, physical activity, socioeconomic status, and healthcare access can influence the distribution of obesity indicators, blood pressure, and the strength of associations between obesity indicators and incident hypertension. For instance, the average 24-h urinary sodium excretion in rural areas is significantly higher than that in urban residents (44), which indicates a higher sodium intake in rural areas, and high sodium intake is significantly associated with greater BMI and larger WC (45). Second, the median follow-up time in our cohort was relatively short (~4 years). Hypertension is a chronic, cumulative disease and the influence of adiposity and body-shape changes on incident hypertension may evolve over longer time horizons. Consequently, our estimates primarily reflect short-to-medium term associations and should be interpreted with caution when considering long-term (e.g., 10-year) risk prediction. Third, the Mendelian randomization analysis was limited to four obesity indicators (BMI, WC, WHR, and BFP), because large-scale GWAS summary statistics with sufficient genome-wide significant SNPs were only available for these traits. For other obesity-related indicators, suitable genetic instruments are currently lacking. Lastly, the study focused solely on adults in Central China, cautioning against generalizing findings to other populations. Despite these limitations, the large cohort size enables a thorough exploration of varying associations between different obesity indicators and hypertension incidence. Additionally, in MR analysis, confounding due to mediated effects was acknowledged and should be considered, suggesting future research should explore these and other effect-modifying factors to deepen understanding of observed relationships.

## 5 Conclusion

Different obesity indicators were significantly associated with the risk of new-onset hypertension, with BMI potentially exhibiting the strongest predictive ability. While overweight and obesity may extend total LE, they are associated with a shorter HALE. Furthermore, this study provides clear evidence of a positive causal relationship between obesity indicators and hypertension, offering a theoretical basis for the early screening and prevention of hypertension in rural areas.

## Data Availability

The original contributions presented in the study are included in the article/Supplementary material, further inquiries can be directed to the corresponding author.
