# Supplementary material for: Evaluating and comparing the predictive ability with hypertension risk among obesity indicators: a prospective cohort integrating Mendelian randomization analysis
Source: Front Nutr. 2025 Dec 5;12:1660842. doi: 10.3389/fnut.2025.1660842 (PMC12715603; doi:10.3389/fnut.2025.1660842)
Supplement: Supplementary file 1 [file Data_Sheet_1.docx]

**Text S1. The sample size calculation and power analysis**

To ensure adequate statistical power, three complementary approaches was employed for sample size estimation.

Firstly, the range of relative risk (*RR*) is from 1.08 to 1.34 to different obesity indicators, and the corresponding sample size is from 1773 to 2286 subjects under the condition of α=0.05 and β=0.10. The estimated sample size is below the actual study population of this manuscript.

Secondly, sensitivity analyses with varying sample sizes showed that the model’s AUC stabilized at around 4,000 participants, confirming that the current sample size is sufficient for robust model performance.

Thirdly, the Hanley-McNeil method as a gold-standard approach for sample size estimation and power analysis, it is often used to assess the sample size and power analysis in AUC-based model validation. It normalizes AUC values to z-scores, enabling calculation based on the statistical framework of mean difference tests. The analysis was guided by three core parameters, as follows:

**① Minimum Clinically Relevant AUC (AUC_0_):** Set to 0.70. This threshold is widely accepted in hypertension prediction as the minimum AUC for a model to have practical utility-models with AUC < 0.70 fail to reliably distinguish between positive and negative outcomes.

**② Expected AUC of the Proposed Model (AUC_1_)**: Set to 0.82. This value was derived from two sources: the model’s performance in the development cohort (AUC = 0.844, 95% CI: 0.835–0.853).

**③ Statistical Parameters:**

Significance level (α): 0.05 (two-tailed), consistent with standard conventions for model validation.

Desired power (1-β): 0.80 (80%), ensuring a low risk of Type II errors (failing to detect a clinically meaningful AUC if it exists).

The sample estimation was conducted using the Hanley-McNeil method. The sample size for this experiment was 172 new-onset subjects with hypertension, and the estimated sample size is below the actual newly diagnosed patients with hypertension of this manuscript (n=1655).

**
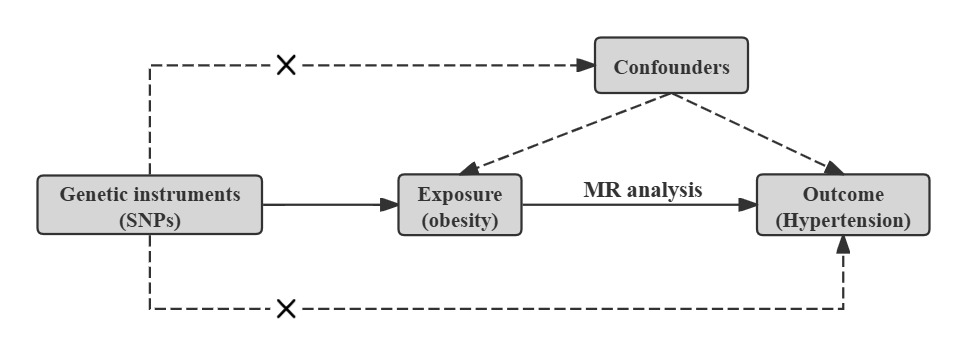
**

**Figure S1. The illustrative diagram of two-sample Mendelian randomization analysis.**

**
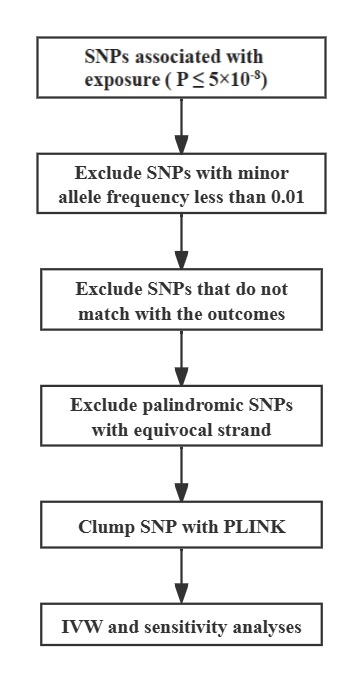
**

**Figure S2. Flow chart of this Mendelian randomization study.**


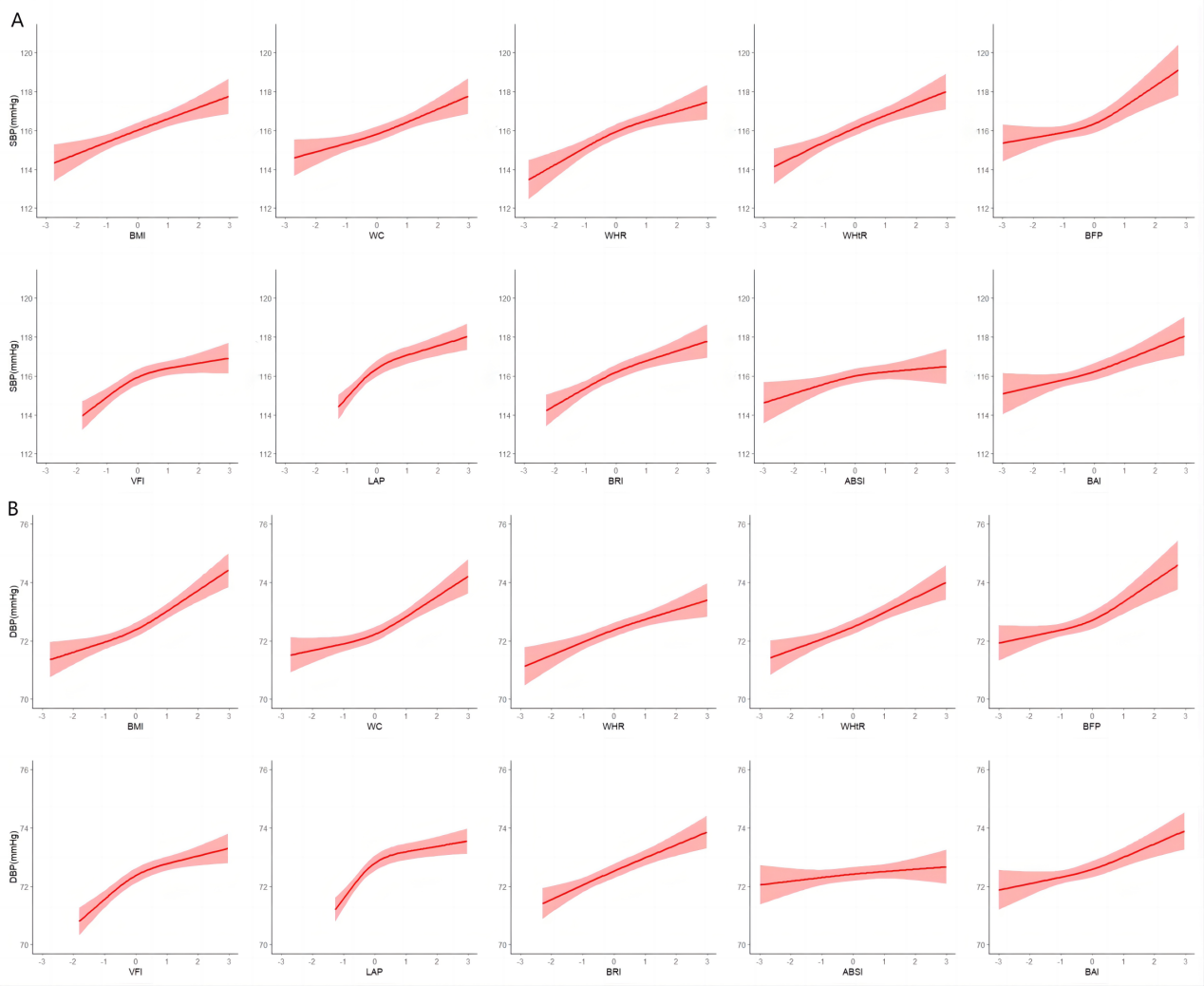


**Figure S3.** The dose-response relationship between standardized obesity indicators and follow-up blood pressure. A fully adjusted models included age, gender, monthly income, baseline systolic blood pressure, baseline diastolic blood pressure, physical activity, vegetables and fruit intake, and family history of hypertension. A is the relationship between different obesity indicators and systolic blood pressure, and B is the relationship between different obesity indicators and diastolic blood pressure. WC: waist circumstance; BMI: body mass index; BFP: body fat percentage; VFI: visceral fat index; WHR: waist-hip ratio; WHtR: waist- to-height ratio; LAP: lipid accumulation product; BRI: body roundness index; ABSI: A body shape index; BAI: body adiposity index


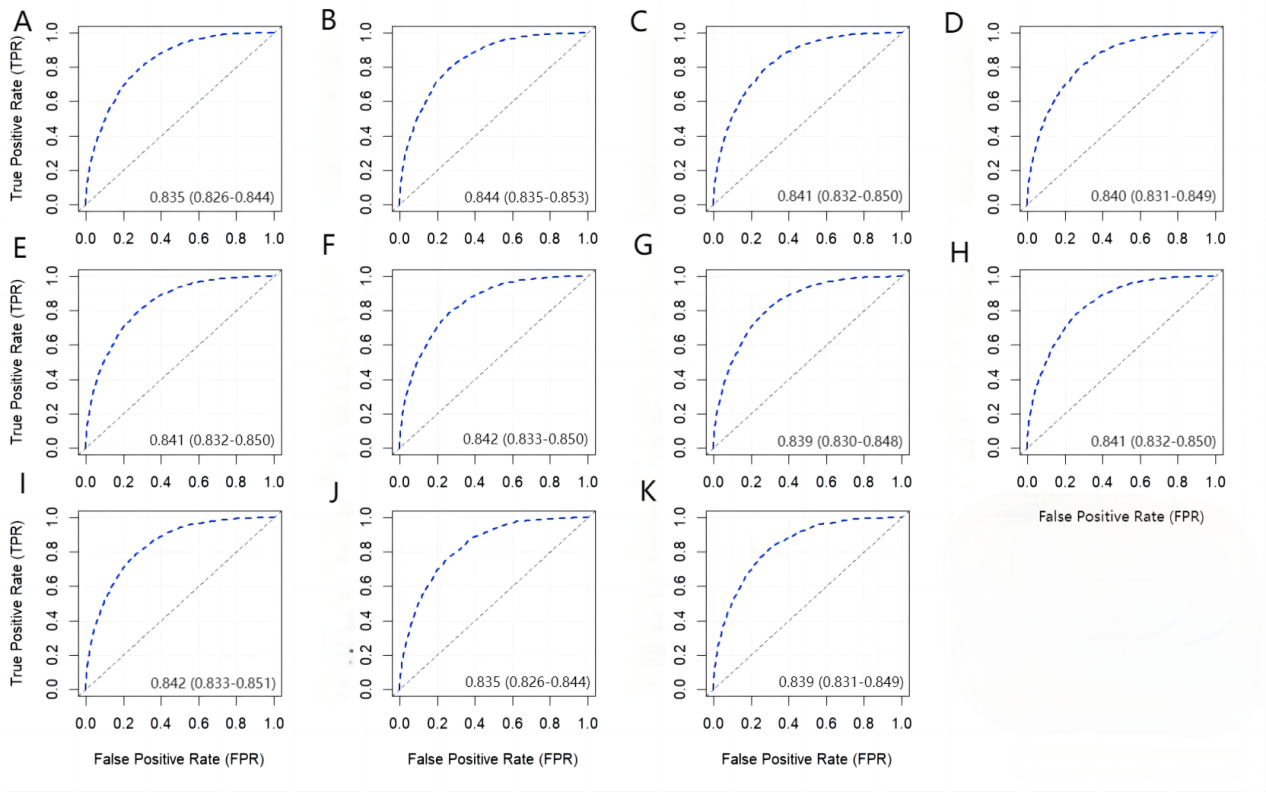


**Figure S4.** ROC of predictive models established by GBM. A: basic model; B: basic model+BMI; C: basic model+WC; D: basic model+WHR; E: basic model+WHtR; F: basic model+BFP; G:basic model+VFI; H: basic model+LAP; I:basic model+BRI; J:basic model+ABSI; K: basic model+BAI. ROC: receiver operating characteristic curve; GBM, gradient boosting machine.
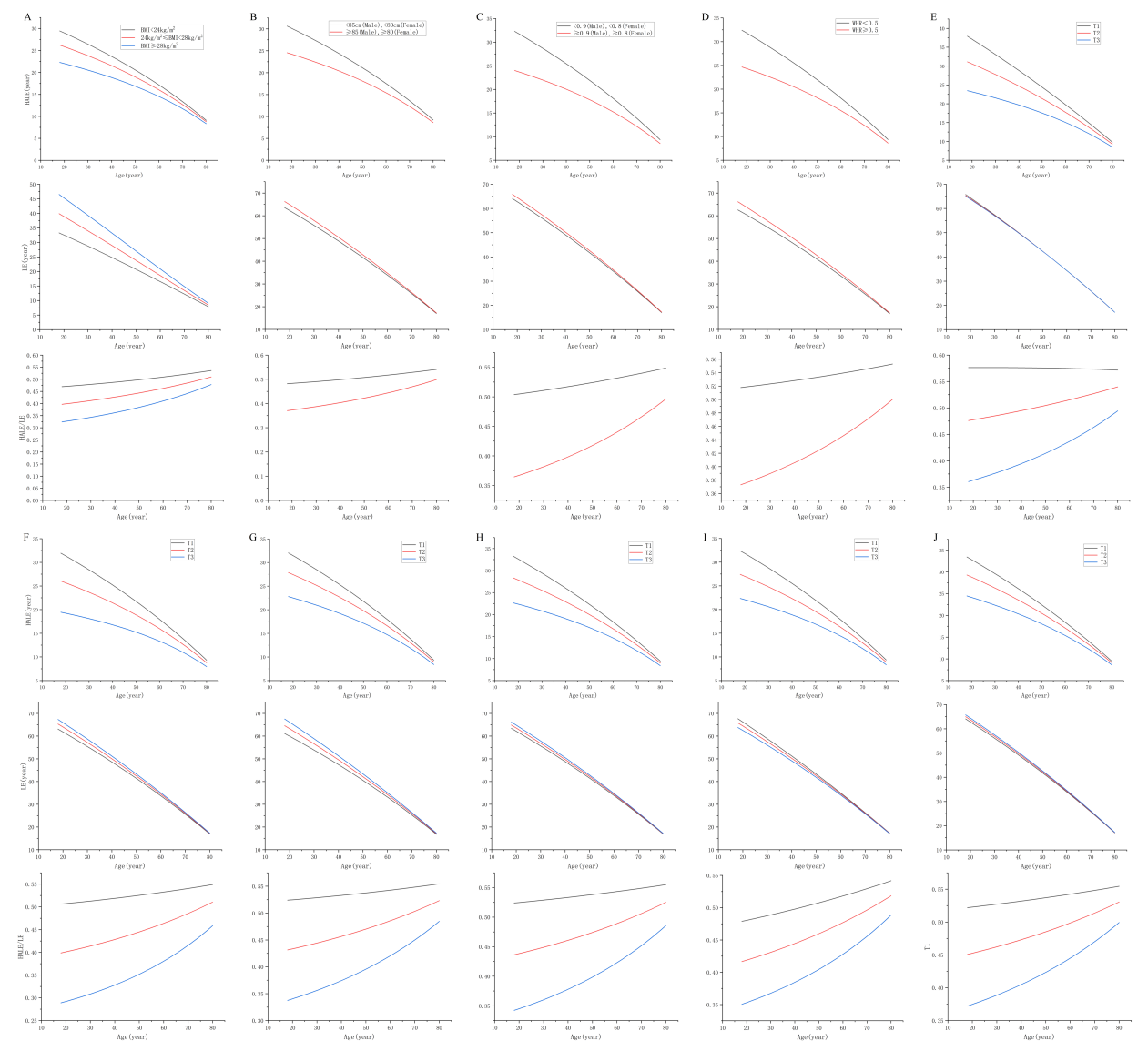


**Figure S5.** Estimates of life expectancy among different obesity indicators groups. A: estimates of life expectancy among different BMI groups; B: estimates of life expectancy among different WC groups; C: estimates of life expectancy among different WHR groups; D: estimates of life expectancy among different WHtR groups; E: estimates of life expectancy among different BFP groups; F: estimates of life expectancy among different VFI groups; G: estimates of life expectancy among different LAP groups; H: estimates of life expectancy among different BRI groups; I: estimates of life expectancy among different ABSI groups; J: estimates of life expectancy among different BAI groups.

| **Table S1. Characteristics of the seven genome-wide association studies used in the present work.** | | | | | | |
| --- | --- | --- | --- | --- | --- | --- |
| **Variable** | **N** | **No. SNPs** | **Year** | **Population** | **Consortium*** | **Source** |
| **Exposure variables: anthropometric traits** | | | | | | |
| **BMI** | **681,275** | **2,336,260** | **2018** | **European** | **GIANT** | **PubMed ID:30124842** |
| **WC** | **407,661** | **10,783,687** | **2021** | **European** | **UK Biobank** | **PubMed ID: 34017140** |
| **WHR** | **502,773** | **11,973,122** | **2018** | **European** | **UK Biobank** | **PubMed ID: 29892013** |
| **BFP** | **331,117** | **10,894,596** | **2017** | **European** | **Neale Lab** | **IEU OpenGWAS project (ukb-a-264)** |
| **BMI** | **158,284** | **5,961,390** | **2017** | **East Asian** | **---** | **PubMed ID: 28892062** |
| **Outcome variables** | | | | | | |
| **HTN** | **54,358 cases，**  **408,652 controls** | **9,851,867** | **2018** | **European** | **MRC-IEU** | **IEU OpenGWAS project (ukb-b-12493)** |
| **SBP** | **340,159** | **19,051,638** | **2021** | **European** | **UK Biobank** | **PubMed ID:34594039** |
| **DBP** | **340,162** | **19,055,470** | **2021** | **European** | **UK Biobank** | **PubMed ID:34594039** |
| **HTN** | **3,903 cases，**  **2,607 controls** | **9,313,379** | **2024** | **East Asian** | **---** | **PubMed ID: 39024449** |

| **Table S2.** Comparison of obesity indicators of participants according to hypertension. | | | | |
| --- | --- | --- | --- | --- |
| **Obesity Indicators** | **Total**  **(n=22, 912)** | **Hypertension**  **(n=1, 655)** | **non-hypertension**  **(n=21, 257)** | ***P* value** |
| BMI (kg/m2, mean±SD) | 24.25 ± 3.34 | 25.03 ± 3.39 | 24.19 ± 3.33 | <0.001 |
| **WC (cm**, mean±SD**)** | 82.20 ± 9.88 | 84.93 ± 9.85 | 81.98 ± 9.85 | <0.001 |
| **WHR (**mean±SD**)** | 0.88 ± 0.07 | 0.90 ± 0.07 | 0.87 ± 0.07 | <0.001 |
| **WHtR (**mean±SD**)** | 0.51 ± 0.06 | 0.54 ± 0.06 | 0.51 ± 0.06 | <0.001 |
| **BFP (**mean±SD**)** | 29.07 ± 6.59 | 30.75 ± 6.28 | 28.94 ± 6.60 | <0.001 |
| **VFI (**mean±SD**)** | 8.51 ± 4.16 | 9.82 ± 4.30 | 8.41 ± 4.13 | <0.001 |
| **LAP (**mean±SD**)** | 36.67 ± 35.23 | 46.65 ± 42.91 | 35.90 ± 34.44 | <0.001 |
| **BRI (**mean±SD**)** | 3.71 ± 1.22 | 4.13 ± 1.25 | 3.67 ± 1.21 | <0.001 |
| **ABSI (**mean±SD**)** | 0.08 ± 0.01 | 0.08 ± 0.01 | 0.08 ± 0.01 | <0.001 |
| **BAI (**mean±SD**)** | 28.41 ± 4.11 | 29.29 ± 4.24 | 28.35 ± 4.09 | <0.001 |
| **Notes:** Data were presented as n (%) or mean **±** standard deviation (SD), as appropriate.  **Abbreviations:** BMI, body mass index; WC, WHR, waist-hip ratio; WHtR, waist circumstance; BFP, body fat percentage; VFI, visceral fat index; waist- to-height ratio; LAP, lipid accumulation product; BRI, body roundness index; ABSI, A body shape index; BAI, body adiposity index. | | | | |

| **Table S3.** The cutoff values of different obesity indicators. | | | |
| --- | --- | --- | --- |
| **Variable** | **T1** | **T2** | **T3** |
| **BFP** | 26.40 | 32.70 | 59.50 |
| **VFI** | 6.00 | 10.00 | 27.00 |
| **LAP** | 18.48 | 38.66 | 455.90 |
| **BRI** | 3.10 | 4.14 | 11.21 |
| **ABSI** | 0.07 | 0.08 | 0.16 |
| **BAI** | 26.47 | 30.07 | 47.63 |
| **Abbreviations:** BFP, body fat percentage; VFI, visceral fat index; waist- to-height ratio; LAP, lipid accumulation product; BRI, body roundness index; ABSI, A body shape index; BAI, body adiposity index. | | | |

| **Table S4**. NRI, IDI and Brier score of predictive models established by GBM. | | | |
| --- | --- | --- | --- |
| Model | NRI | IDI | Brier score |
| Basic model + BMI | 0.0292 | 0.0043 | 0.085 |
| Basic model + WC | 0 | 0.0022 | 0.127 |
| Basic model + WHR | 0.0071 | 0.0046 | 0.090 |
| Basic model + WHtR | 0.0189 | 0.0026 | 0.089 |
| Basic model + BFP | 0.0342 | 0.0050 | 0.086 |
| Basic model + VFI | 0.0203 | 0.0018 | 0.107 |
| Basic model + LAP | 0.0029 | 0.0065 | 0.119 |
| Basic model + BRI | 0.0019 | 0.0022 | 0.120 |
| Basic model + ABSI | -0.0135 | 0.0020 | 0.122 |
| Basic model + BAI | -0.0068 | 0.0022 | 0.121 |
| Abbreviations: AUC, area under the curve. | | | |

| **Table S5.** AUC of predictive models established by GBM among participants under 60 years old. | | |
| --- | --- | --- |
| **Model** | **AUC** | **95% *CI*** |
| **Basic model** | 0.876 | 0.865-0.887 |
| **Basic model + BMI** | 0.882 | 0.871-0.893 |
| **Basic model + WC** | 0.881 | 0.870-0.892 |
| **Basic model + WHR** | 0.880 | 0.869-0.890 |
| **Basic model + WHtR** | 0.877 | 0.866-0.888 |
| **Basic model + BFP** | 0.882 | 0.871-0.893 |
| **Basic model + VFI** | 0.879 | 0.869-0.890 |
| **Basic model + LAP** | 0.886 | 0.875-0.896 |
| **Basic model + BRI** | 0.882 | 0.871-0.893 |
| **Basic model + ABSI** | 0.882 | 0.871-0.892 |
| **Basic model + BAI** | 0.884 | 0.873-0.895 |
| Abbreviations: AUC, area under the curve; GBM, gradient boosting machine; BMI, body mass index; WC, WHR, waist-hip ratio; WHtR, waist circumstance; BFP, body fat percentage; VFI, visceral fat index; waist- to-height ratio; LAP, lipid accumulation product; BRI, body roundness index; ABSI, A body shape index; BAI, body adiposity index | | |

| **Table S6.** AUC of predictive models established by GBM among participants over 60 years old. | | |
| --- | --- | --- |
| **Model** | **AUC** | **95% *CI*** |
| **Basic model** | 0.825 | 0.811-0.839 |
| **Basic model + BMI** | 0.847 | 0.833-0.860 |
| **Basic model + WC** | 0.837 | 0.823-0.851 |
| **Basic model + WHR** | 0.835 | 0.821-0.849 |
| **Basic model + WHtR** | 0.833 | 0.819-0.847 |
| **Basic model + BFP** | 0.843 | 0.829-0.856 |
| **Basic model + VFI** | 0.837 | 0.823-0.851 |
| **Basic model + LAP** | 0.836 | 0.822-0.850 |
| **Basic model + BRI** | 0.837 | 0.823-0.851 |
| **Basic model + ABSI** | 0.829 | 0.815-0.843 |
| **Basic model + BAI** | 0.834 | 0.820-0.848 |
| Abbreviations: AUC, area under the curve; GBM, gradient boosting machine; BMI, body mass index; WC, WHR, waist-hip ratio; WHtR, waist circumstance; BFP, body fat percentage; VFI, visceral fat index; waist- to-height ratio; LAP, lipid accumulation product; BRI, body roundness index; ABSI, A body shape index; BAI, body adiposity index | | |

| **Table S7.** AUC of predictive models established by GBM among male. | | |
| --- | --- | --- |
| **Model** | **AUC** | **95% *CI*** |
| **Basic model** | 0.861 | 0.848-0.875 |
| **Basic model + BMI** | 0.873 | 0.860-0.887 |
| **Basic model + WC** | 0.874 | 0.860-0.887 |
| **Basic model + WHR** | 0.872 | 0.859-0.886 |
| **Basic model + WHtR** | 0.867 | 0.853-0.881 |
| **Basic model + BFP** | 0.875 | 0.862-0.888 |
| **Basic model + VFI** | 0.871 | 0.857-0.884 |
| **Basic model + LAP** | 0.873 | 0.860-0.886 |
| **Basic model + BRI** | 0.869 | 0.856-0.883 |
| **Basic model + ABSI** | 0.865 | 0.851-0.879 |
| **Basic model + BAI** | 0.866 | 0.852-0.879 |
| Abbreviations: AUC, area under the curve; GBM, gradient boosting machine; BMI, body mass index; WC, WHR, waist-hip ratio; WHtR, waist circumstance; BFP, body fat percentage; VFI, visceral fat index; waist- to-height ratio; LAP, lipid accumulation product; BRI, body roundness index; ABSI, A body shape index; BAI, body adiposity index | | |

| **Table S8.** AUC of predictive models established by GBM among female. | | |
| --- | --- | --- |
| **Model** | **AUC** | **95% *CI*** |
| **Basic model** | 0.86 | 0.849-0.870 |
| **Basic model + BMI** | 0.868 | 0.858-0.879 |
| **Basic model + WC** | 0.865 | 0.855-0.875 |
| **Basic model + WHR** | 0.869 | 0.858-0.879 |
| **Basic model + WHtR** | 0.864 | 0.853-0.874 |
| **Basic model + BFP** | 0.866 | 0.855-0.876 |
| **Basic model + VFI** | 0.864 | 0.853-0.875 |
| **Basic model + LAP** | 0.868 | 0.857-0.878 |
| **Basic model + BRI** | 0.868 | 0.858-0.878 |
| **Basic model + ABSI** | 0.866 | 0.856-0.877 |
| **Basic model + BAI** | 0.868 | 0.857-0.878 |
| Abbreviations: AUC, area under the curve; GBM, gradient boosting machine; BMI, body mass index; WC, WHR, waist-hip ratio; WHtR, waist circumstance; BFP, body fat percentage; VFI, visceral fat index; waist- to-height ratio; LAP, lipid accumulation product; BRI, body roundness index; ABSI, A body shape index; BAI, body adiposity index | | |

| **Table S9.** AUC of predictive models established by GBM among participants with non-high-salt diet. | | |
| --- | --- | --- |
| **Model** | **AUC** | **95% *CI*** |
| **Basic model** | 0.844 | 0.834-0.854 |
| **Basic model + BMI** | 0.851 | 0.842-0.867 |
| **Basic model + WC** | 0.854 | 0.844-0.863 |
| **Basic model + WHR** | 0.849 | 0.840-0.859 |
| **Basic model + WHtR** | 0.851 | 0.842-0.861 |
| **Basic model + BFP** | 0.853 | 0.844-0.863 |
| **Basic model + VFI** | 0.850 | 0.841-0.860 |
| **Basic model + LAP** | 0.850 | 0.841-0.859 |
| **Basic model + BRI** | 0.851 | 0.842-0.861 |
| **Basic model + ABSI** | 0.851 | 0.842-0.861 |
| **Basic model + BAI** | 0.85 | 0.840-0.859 |
| Abbreviations: AUC, area under the curve; GBM, gradient boosting machine; BMI, body mass index; WC, WHR, waist-hip ratio; WHtR, waist circumstance; BFP, body fat percentage; VFI, visceral fat index; waist- to-height ratio; LAP, lipid accumulation product; BRI, body roundness index; ABSI, A body shape index; BAI, body adiposity index | | |

| **Table S10.** AUC of predictive models established by GBM among participants with high-salt diet. | | |
| --- | --- | --- |
| **Model** | **AUC** | **95% *CI*** |
| **Basic model** | 0.909 | 0.893-0.925 |
| **Basic model + BMI** | 0.912 | 0.897-0.927 |
| **Basic model + WC** | 0.919 | 0.904-0.933 |
| **Basic model + WHR** | 0.925 | 0.910-0.939 |
| **Basic model + WHtR** | 0.919 | 0.904-0.934 |
| **Basic model + BFP** | 0.911 | 0.894-0.928 |
| **Basic model + VFI** | 0.913 | 0.897-0.928 |
| **Basic model + LAP** | 0.927 | 0.913-0.941 |
| **Basic model + BRI** | 0.911 | 0.895-0.927 |
| **Basic model + ABSI** | 0.921 | 0.906-0.936 |
| **Basic model + BAI** | 0.909 | 0.892-0.926 |
| Abbreviations: AUC, area under the curve; GBM, gradient boosting machine; BMI, body mass index; WC, WHR, waist-hip ratio; WHtR, waist circumstance; BFP, body fat percentage; VFI, visceral fat index; waist- to-height ratio; LAP, lipid accumulation product; BRI, body roundness index; ABSI, A body shape index; BAI, body adiposity index | | |

| **Table S11.** AUC of predictive models established by GBM among participants with low physical activity. | | |
| --- | --- | --- |
| **Model** | **AUC** | **95% *CI*** |
| **Basic model** | 0.885 | 0.872-0.899 |
| **Basic model + BMI** | 0.893 | 0.879-0.906 |
| **Basic model + WC** | 0.898 | 0.885-0.911 |
| **Basic model + WHR** | 0.894 | 0.880-0.908 |
| **Basic model + WHtR** | 0.900 | 0.888-0.913 |
| **Basic model + BFP** | 0.901 | 0.888-0.914 |
| **Basic model + VFI** | 0.895 | 0.881-0.908 |
| **Basic model + LAP** | 0.895 | 0.881-0.908 |
| **Basic model + BRI** | 0.900 | 0.888-0.913 |
| **Basic model + ABSI** | 0.897 | 0.883-0.910 |
| **Basic model + BAI** | 0.897 | 0.883-0.910 |
| Abbreviations: AUC, area under the curve; GBM, gradient boosting machine; BMI, body mass index; WC, WHR, waist-hip ratio; WHtR, waist circumstance; BFP, body fat percentage; VFI, visceral fat index; waist- to-height ratio; LAP, lipid accumulation product; BRI, body roundness index; ABSI, A body shape index; BAI, body adiposity index | | |

| **Table S12.** AUC of predictive models established by GBM among participants with moderate physical activity. | | |
| --- | --- | --- |
| **Model** | **AUC** | **95% *CI*** |
| **Basic model** | 0.857 | 0.844-0.870 |
| **Basic model + BMI** | 0.874 | 0.861-0.886 |
| **Basic model + WC** | 0.869 | 0.857-0.882 |
| **Basic model + WHR** | 0.869 | 0.857-0.882 |
| **Basic model + WHtR** | 0.868 | 0.856-0.881 |
| **Basic model + BFP** | 0.869 | 0.857-0.882 |
| **Basic model + VFI** | 0.860 | 0.847-0.873 |
| **Basic model + LAP** | 0.868 | 0.855-0.880 |
| **Basic model + BRI** | 0.870 | 0.858-0.883 |
| **Basic model + ABSI** | 0.868 | 0.855-0.881 |
| **Basic model + BAI** | 0.868 | 0.855-0.880 |
| Abbreviations: AUC, area under the curve; GBM, gradient boosting machine; BMI, body mass index; WC, WHR, waist-hip ratio; WHtR, waist circumstance; BFP, body fat percentage; VFI, visceral fat index; waist- to-height ratio; LAP, lipid accumulation product; BRI, body roundness index; ABSI, A body shape index; BAI, body adiposity index | | |

| **Table S13.** AUC of predictive models established by GBM among participants with high physical activity. | | |
| --- | --- | --- |
| **Model** | **AUC** | **95% *CI*** |
| **Basic model** | 0.871 | 0.856-0.886 |
| **Basic model + BMI** | 0.888 | 0.873-0.902 |
| **Basic model + WC** | 0.900 | 0.886-0.913 |
| **Basic model + WHR** | 0.887 | 0.873-0.901 |
| **Basic model + WHtR** | 0.887 | 0.873-0.901 |
| **Basic model + BFP** | 0.893 | 0.878-0.907 |
| **Basic model + VFI** | 0.885 | 0.870-0.900 |
| **Basic model + LAP** | 0.886 | 0.872-0.901 |
| **Basic model + BRI** | 0.881 | 0.866-0.896 |
| **Basic model + ABSI** | 0.886 | 0.872-0.901 |
| **Basic model + BAI** | 0.880 | 0.865-0.895 |
| Abbreviations: AUC, area under the curve; GBM, gradient boosting machine; BMI, body mass index; WC, WHR, waist-hip ratio; WHtR, waist circumstance; BFP, body fat percentage; VFI, visceral fat index; waist- to-height ratio; LAP, lipid accumulation product; BRI, body roundness index; ABSI, A body shape index; BAI, body adiposity index | | |

| **Table S14.** Median and Interquartile Range of SHAP Values for Selected Features | | | |
| --- | --- | --- | --- |
| **Obesity Indicators** | **Median SHAP value** | **IQR Lower** | **IQR Lower** |
| BMI | 0.014 | -0.060 | 0.056 |
| WC | -0.010 | -0.048 | 0.035 |
| WHR | 0.000 | -0.145 | 0.139 |
| WHtR | 0.003 | -0.072 | 0.067 |
| BFP | 0.006 | -0.045 | 0.050 |
| VFI | -0.024 | -0.079 | 0.051 |
| LAP | 0.008 | -0.094 | 0.074 |
| BRI | 0.000 | -0.078 | 0.055 |
| ABSI | 0.023 | -0.092 | 0.079 |
| BAI | -0.006 | -0.032 | 0.026 |
| Model adjusted for age, gender, smoking status, more vegetable and fruit intake, family history of hypertension, high-salt diet, SBP and DBP at baseline | | | |

| **Table S15.** Distribution of BMI levels across risk groups predicted by the GBM model. | | |
| --- | --- | --- |
| **Predicted risk group** | **Low-risk** | **High-risk** |
| BMI mean | 23.90 | 25.30 |
| WC mean | 80.70 | 85.60 |
| WHR mean | 0.86 | 0.90 |
| WHtR mean | 0.50 | 0.54 |
| BFP mean | 28.20 | 31.20 |
| VFI mean | 7.75 | 10.00 |
| LAP mean | 31.60 | 48.20 |
| BRI mean | 3.49 | 4.23 |
| ABSI mean | 0.08 | 0.08 |
| BAI mean | 28.00 | 29.40 |
| Abbreviations: GBM, gradient boosting machine; BMI, body mass index; WC, WHR, waist-hip ratio; WHtR, waist circumstance; BFP, body fat percentage; VFI, visceral fat index; waist- to-height ratio; LAP, lipid accumulation product; BRI, body roundness index; ABSI, A body shape index; BAI, body adiposity index. | | |

| **Table S16.** UVMR estimating the associations of obesity indicators with hypertension, SBP and DBP. | | | | | | | |
| --- | --- | --- | --- | --- | --- | --- | --- |
| **Outcome** | **Exposure** | **Method** | **No.of SNPs** | **β** | **OR (95% CI)** | ***P* value** | |
| Hypertension | BFP | Inversevariance weighted | 253 | 0.08 | 1.08 (1.07, 1.09) | 4.67E-41 | |
|  |  | Weighted median | 253 | 0.09 | 1.09 (1.08, 1.10) | 9.86E-56 | |
|  |  | MR Egger | 253 | 0.04 | 1.04 (1.00, 1.09) | 0.0335 | |
|  |  | MR PRESSO | 23* |  |  | 3.87E-50 | |
|  | BMI | Inverse variance weighted | 492 | 0.06 | 1.06 (1.06, 1.07) | 4.93E-94 | |
|  |  | Weighted median | 492 | 0.06 | 1.06 (1.05, 1.07) | 2.25E-56 | |
|  |  | MR Egger | 492 | 0.05 | 1.06 (1.04, 1.07) | 7.25E-11 | |
|  |  | MR PRESSO | 17* |  |  | 2.88E-81 | |
|  | WC | Inverse variance weighted | 245 | 0.08 | 1.08 (1.07, 1.09) | 4.90E-71 | |
|  |  | Weighted median | 245 | 0.07 | 1.08 (1.06, 1.09) | 6.93E-31 | |
|  |  | MR Egger | 245 | 0.07 | 1.07 (1.04, 1.10) | 7.42E-08 | |
|  |  | MR PRESSO | 7* |  |  | 1.50E-55 | |
|  | WHR | Inverse variance weighted | 314 | 0.03 | 1.03 (1.02, 1.04) | 8.35E-15 | |
|  |  | Weighted median | 314 | 0.03 | 1.03 (1.02, 1.04) | 2.51E-14 | |
|  |  | MR Egger | 314 | 0.04 | 1.04 (1.02, 1.06) | 2.28E-05 | |
|  |  | MR PRESSO | 22* |  |  | 2.43E-23 | |
| SBP | BFP | Inverse variance weighted | 252 | 0.16 | 1.18 (1.13, 1.23) | 3.27E-14 | |
|  |  | Weighted median | 252 | 0.18 | 1.20 (1.16, 1.24) | 1.02E-24 | |
|  |  | MR Egger | 252 | 0.02 | 1.02 (0.88, 1.19) | 0.7878 | |
|  |  | MR PRESSO | 37* |  |  | 2.86E-28 | |
|  | BMI | Inverse variance weighted | 480 | 0.13 | 1.14 (1.11, 1.17) | 1.38E-27 | |
|  |  | Weighted median | 480 | 0.14 | 1.15 (1.12, 1.18) | 6.14E-30 | |
|  |  | MR Egger | 480 | 0.11 | 1.12 (1.05, 1.19) | 0.0003 | |
|  |  | MR PRESSO | 43* |  |  | 1.37E-43 | |
|  | WC | Inverse variance weighted | 279 | 0.14 | 1.15 (1.11, 1.19) | 4.46E-16 | |
|  |  | Weighted median | 279 | 0.17 | 1.19 (1.15, 1.23) | 1.39E-26 | |
|  |  | MR Egger | 279 | 0.11 | 1.12 (1.02, 1.24) | 0.0235 | |
|  |  | MR PRESSO | 33* |  |  | 1.94E-32 | |
|  | WHR | Inverse variance weighted | 310 | 0.06 | 1.07 (1.04, 1.10) | 3.79E-06 | |
|  |  | Weighted median | 310 | 0.07 | 1.07 (1.04, 1.09) | 1.34E-07 | |
|  |  | MR Egger | 310 | 0.05 | 1.05 (0.98, 1.13) | 0.1395 | |
|  |  | MR PRESSO | 37* |  |  | 1.96E-12 | |
| DBP | BFP | Inverse variance weighted | 252 | 0.09 | 1.24 (1.18, 1.29) | 7.19E-20 | |
|  |  | Weighted median | 252 | 0.14 | 1.23 (1.18, 1.28) | 5.69E-26 | |
|  |  | MR Egger | 252 | 0.13 | 1.13 (0.96, 1.33) | 0.1545 | |
|  |  | MR PRESSO | 35* |  |  | 2.81E-34 | |
|  | BMI | Inverse variance weighted | 483 | 0.11 | 1.20 (1.17, 1.23) | 2.68E-47 | |
|  |  | Weighted median | 483 | 0.10 | 1.17 (1.14, 1.20) | 2.85E-37 | |
|  |  | MR Egger | 483 | 0.12 | 1.13 (1.06, 1.20) | 0.0002 | |
|  |  | MR PRESSO | 37* |  |  | 5.50E-66 | |
|  | WC | Inverse variance weighted | 281 | 0.12 | 1.20 (1.16, 1.24) | 1.82E-26 | |
|  |  | Weighted median | 281 | 0.10 | 1.18 (1.15, 1.22) | 1.67E-23 | |
|  |  | MR Egger | 281 | 0.05 | 1.13 (1.03, 1.24) | 0.0137 | |
|  |  | MR PRESSO | 27* |  |  | 2.03E-39 | |
|  | WHR | Inverse variance weighted | 310 | 0.04 | 1.05 (1.02, 1.08) | 0.0031 | |
|  |  | Weighted median | 310 | 0.00 | 1.06 (1.03, 1.09) | 2.67E-05 | |
|  |  | MR Egger | 310 | 0.01 | 1.06 (0.98, 1.14) | 0.1381 | |
|  |  | MR PRESSO | 43* |  |  | 5.82E-06 | |
| Hypertension | BMI | Inverse variance weighted | 261 | 0.36 | 1.44 (1.07, 1.93) | 0.02 |  |
|  |  | Weighted median | 261 | -0.07 | 0.93 (0.59, 1.48) | 0.76 | |
|  |  | MR Egger | 261 | 0.55 | 1.75 (0.70, 4.37) | 0.23 | |
| * NO. of outliers.  Abbreviations: CI, confidence interval; IVW, inverse variance weighted; MR, Mendelian randomization; OR, odds ratio; PRESSO, pleiotropy residual sum and outlier; SNP, single nucleotide polymorphism; UVMR, univariable Mendelian randomization | | | | | | | |

| **Table S17**. MR heterogeneity test of the associations of obesity indicators with hypertension, SBP and DBP. | | | | | |
| --- | --- | --- | --- | --- | --- |
| **Outcome** | **Exposure** | **Method** | **Q** | **Q df** | **Q *p*-value** |
| Hypertension | BFP | MR Egger | 858.30 | 251 | 2.02E-67 |
|  |  | Inverse variance weighted | 868.22 | 252 | 1.10E-68 |
|  | BMI | MR Egger | 1052.24 | 490 | 3.76E-43 |
|  |  | Inverse variance weighted | 1055.23 | 491 | 2.47E-43 |
|  | WC | MR Egger | 508.38 | 243 | 6.89E-21 |
|  |  | Inverse variance weighted | 510.21 | 244 | 6.18E-21 |
|  | WHR | MR Egger | 934.76 | 312 | 2.05E-63 |
|  |  | Inverse variance weighted | 938.86 | 313 | 9.02E-64 |
|  | BFP | MR Egger | 1394.76 | 250 | 4.25E-158 |
|  |  | Inverse variance weighted | 1415.28 | 251 | 2.16E-161 |
|  | BMI | MR Egger | 1840.83 | 478 | 9.47E-159 |
|  |  | Inverse variance weighted | 1842.31 | 479 | 1.07E-158 |
|  | WC | MR Egger | 1258.85 | 277 | 6.87E-125 |
|  |  | Inverse variance weighted | 1260.65 | 278 | 7.24E-125 |
|  | WHR | MR Egger | 1463.85 | 308 | 1.56E-149 |
|  |  | Inverse variance weighted | 1464.74 | 309 | 2.39E-149 |
| DBP | BFP | MR Egger | 1425.95 | 250 | 1.11E-163 |
|  |  | Inverse variance weighted | 1433.47 | 251 | 1.19E-164 |
|  | BMI | MR Egger | 1819.82 | 481 | 1.67E-154 |
|  |  | Inverse variance weighted | 1835.14 | 482 | 1.15E-156 |
|  | WC | MR Egger | 1092.21 | 279 | 1.44E-96 |
|  |  | Inverse variance weighted | 1099.66 | 280 | 1.77E-97 |
|  | WHR | MR Egger | 1615.11 | 308 | 7.41E-176 |
|  |  | Inverse variance weighted | 1615.62 | 309 | 1.38E-175 |
| Hypertension | BMI | MR Egger | 277.657 | 259 | 0.203 |
|  |  | Inverse variance weighted | 277.873 | 260 | 0.213 |
| Abbreviations: df, degree of freedom; IVW, inverse variance weighted; MR, Mendelian randomization. | | | | | |

| **Table S18.** MR directional pleiotropy test (MR Egger) of the associations of obesity indicators with hypertension, SBP and DBP. | | | | |
| --- | --- | --- | --- | --- |
| **Outcome** | **Exposure** | **Egger intercept** | **SE** | ***P* value** |
| Hypertension | BFP | 0.0005 | 0.0003 | 0.09 |
|  | BMI | 0.0002 | 0.0001 | 0.24 |
|  | WC | 0.0002 | 0.0002 | 0.35 |
|  | WHR | -0.0002 | 0.0002 | 0.24 |
| SBP | BFP | 0.0023 | 0.0012 | 0.06 |
|  | BMI | 0.0003 | 0.0005 | 0.54 |
|  | WC | 0.0005 | 0.0008 | 0.53 |
|  | WHR | 0.0003 | 0.0007 | 0.66 |
| DBP | BFP | 0.0015 | 0.0013 | 0.25 |
|  | BMI | 0.0011 | 0.0005 | 0.04 |
|  | WC | 0.0011 | 0.0008 | 0.17 |
|  | WHR | -0.0002 | 0.0008 | 0.75 |
| Hypertension | BMI | -0.00495 | 0.011 | 0.654 |
| Abbreviations: MR, Mendelian randomization; SE, standard error. | | | | |
